# Intraoral scanners in children: evaluation of the patient perception, reliability and reproducibility, and chairside time—A systematic review

**DOI:** 10.3389/fped.2023.1213072

**Published:** 2023-06-26

**Authors:** Diego Serrano-Velasco, Andrea Martín-Vacas, Marta M. Paz-Cortés, Giovanni Giovannini, Patricia Cintora-López, Juan Manuel Aragoneses

**Affiliations:** ^1^ PhD Program in Translational Medicine, San Pablo CEU University, Madrid, Spain; ^2^ Faculty of Dentistry, Alfonso X El Sabio University, Madrid, Spain; ^3^ Master in Paediatric Dentistry, Faculty of Dentistry, Complutense University of Madrid, Madrid, Spain; ^4^ Department of Dental Research, Federico Henriquez y Carvajal University, Santo Domingo, Dominican Republic

**Keywords:** intraoral scanner, digital flow, orthodontic, dental practices, pediatric dentistry

## Abstract

**Purpose:**

The aim of this systematic review is to evaluate the perception of the patient, the chairside time, and the reliability and/or reproducibility of intraoral scanners for full arch in pediatric patients.

**Methods:**

A data search was performed in four databases (Medline-Pubmed, Scopus, ProQuest and Web of Science) in accordance with the PRISMA 2020 statements. Studies were classified in three categories (patient perception, scanning or impression time and reliability and/or reproducibility). The resources, the data extraction and the quality assessment were carried out independently by two operators. The variables recorded were population characteristics, material and methods aspects and included country, study design and main conclusion. A quality assessment of the selected studies was performed with QUADAS-2 tool, and Kappa-Cohen Index was calculated to analyze examiner agreement.

**Results:**

The initial search obtained 681 publications, and finally four studies matching inclusion criteria were selected. The distribution of the studies in the categories was three for the analysis of the patient's perception and scanning or impression time; and two items to assess the reliability and/or reproducibility of intraoral scans. All included studies have a repeated measures–transversal design. The sample size ranged between 26 and 59 children with a mean age. The intraoral scanners evaluated were Lava C.O.S, Cerec Omnicam, TRIOS Classic, TRIOS 3-Cart and TRIOS Ortho. The quality assessment of the studies using QUADAS-2 tool revealed a low risk of bias while evaluating patient perception, but an unclear risk of bias in the analysis of accuracy or chairside time. In relation to the applicability concerns, the patient selection was of high risk of bias. All studies agreed that the patient perception and comfort is better with intraoral scanners in comparison with the conventional method. The accuracy or reliability of the digital procedure is not clear, being clinically acceptable. In relation with the chairside time, it depends on the intraoral scanner, with contradictory data in the different analyzed studies.

**Conclusion:**

The use of intraoral scanners in children is a favorable option, finding a significantly higher patient perception and comfort with intraoral scanners compared to the conventional impression method. The evidence for reliability or reproducibility is not strong to date, however, the differences between the intraoral measurements and the digital models would be clinically acceptable.

## 1. Introduction

The reproduction of intraoral structures using impression techniques is a common procedure in the dental clinic, since it is required for multiple procedures, the most common prosthodontics and orthodontics. In the field of orthodontics, full-arch impressions are used for diagnostic purposes, treatment plan development, and appliance fabrication. The conventional impression procedure is highly sensitive to the technique, and the precision of the models can be altered in relation to the materials used, presence of artifacts, material compression, casting time, and ambient temperature (1).

The concept of intraoral scanner was introduced in dentistry in the 1970 s (2), and the first orthodontic scanner system emerged in 1999 developed by Cadent Technologies Inc. (OrthoCAD). Since then, a wide market for scanning technology was opened, and there are currently various systems available. Obtaining digital models using intraoral scanners has been gaining popularity over the years, due to its multiple advantages. Among the benefits of intraoral scanning systems are real-time visualization, ease and selectivity of repetition, selective capture of areas of interest, shorter disinfection time than conventional impressions, image analysis options, absence of model wear, ease of archiving and data fusion, among others (1, 3). In addition, patients report that the perception and comfort of the intraoral scanner is better than with conventional impressions (4, 5). However, the intraoral scanners have some limitations such as the existence of a higher learning curve, the inability to modify the occlusion of the patient, the high costs of the system and the rigid sequence of scanning (3). Several studies have been carried out that analyze the reliability and reproducibility of intraoral scanners. Although the studies seem to offer positive results regarding the validity of intraoral scanners, the difference in study methodology and the different intraoral scanners on the market make the evidence inconclusive (6, 7).

Currently, children make up a large part of the volume of orthodontic patients, who, due to their behavioral characteristics and the presence of smaller oral structures, can compromise both conventional and intraoral dental impression procedures. There are studies that analyze the time needed, the and the reliability of intraoral scanners in children (8–12), and even studies that have come to manufacture space maintainers through digital models (13, 14); however, the quality of the evidence is not known and therefore it is difficult to draw a strong conclusion on the use of intraoral scanners in children. The aim of this systematic review is to evaluate the perception of the patient, the chairside time, and the reliability and/or reproducibility of intraoral scanners for full arch in pediatric patients.

## 2. Materials and methods

This systematic review was conducted in accordance with the Preferred Reporting Items for Systematic Reviews and Meta-Analyses (PRISMA) 2020 statement (15).

### 2.1. Eligibility criteria

**Figure 1:**
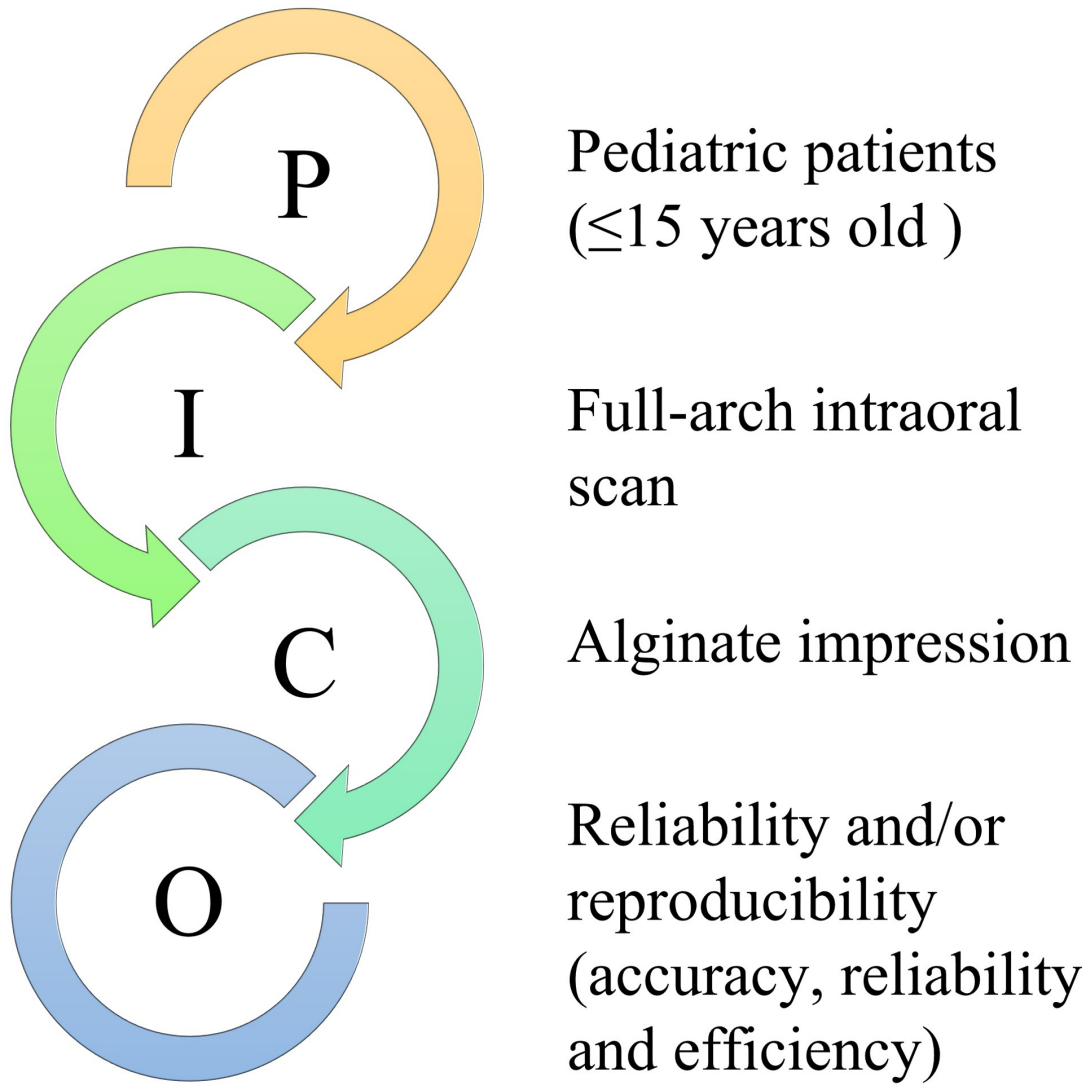
PICO question used for the study selection.

PICO question (Figure 1) was formulated for study selection. Studies conducted in pediatric patients were included, that is, age ≤15 years old (population), who underwent a full-arch intraoral scan (intervention) and an alginate impression to obtain plaster models (comparison) with the aim of to analyze the reliability and/or reproducibility of the procedures (outcome). The reliability and reproducibility will correspond to criteria of accuracy, reliability, and efficiency (6). The inclusion criteria established for the selection of articles were analytical studies in English or Spanish. Literature reviews, systematic reviews, meta-analyses, and observational studies were excluded. Only studies with direct intraoral scanning and measuring of the patient were included, not on stone models (*in vitro*), to avoid procedural bias.

### 2.2. Search strategy

The data search was carried out the 11th of December 2022 in the Medline (PubMed), Scopus, ProQuest, and Web of Science (WOS) databases, using the keywords “*digital impression, intraoral digital impression, intraoral scanning, intraoral scanner, intraoral digital scanner, child and referrals and pediatric*”, excluding “*adults*”. No time restrictions were applied. The search strategies recommended in each of the databases were used. In addition, a “snowball” search of the reference list of the articles selected for full-text reading was carried out, with the aim of identifying additional studies.

### 2.3. Study selection and data extraction

The identification and screening procedure was carried out by two different reviewers (DS-V and AM-V) independently, coming together at the end of the procedure and carrying out the final inclusion of articles. During the procedure, PRISMA guidelines were applied. In case of disagreement, consensus was reached by discussion of the criteria between the reviewers. For data extraction, a table was prepared specifying data on the study population (sample size, age, and inclusion and exclusion criteria), material and methods (scanner evaluated and compared, parameters and measurements collected, and statistical test performed) and the final conclusions of each article. The articles selected for the systematic review were ordered into three categories depending on the variables evaluated, with the objective of knowing the quality of the studies in each aspect, these being (1) patient perception, (2) scanning or impression time and (3) reliability and/or accuracy.

The perception and comfort of the patient were assessed using different scales, so it was not possible to measure the effect. Quantitative measures (time and reliability and/or reproducibility) could also not be analyzed as study methodology differed significantly between studies.

### 2.4. Assessment of quality

The quality of the articles was assessed using the Quality Assessment of Diagnostic Accuracy Studies-2 (QUADAS-2) tool (16, 17) independently in the three thematic groups studied, which analyses the risk of bias (patient selection, index test, reference standard and flow and timing) and applicability concerns (patient selection, index test and reference standard) classifying each item as high, low, or not determined. QUADAS-2 is the current version of QUADAS and the tool recommended for the use in systematic reviews to evaluate the risk of bias and applicability of primary diagnostic accuracy studies. It consists of four key domains: patient selection, index test, reference standard, and flow and timing. Each domain is assessed in terms of risk of bias, and the first three in terms of concerns regarding applicability. It includes signaling questions to assist in judgements, applied in four phases: (1) summarize the review question, (2) tailor the tool to the review and produce review-specific guidance, (3) construct a flow diagram for the primary study and (4) assess risk of bias and concerns regarding applicability. Signaling questions consider applicability of the results, and questions about the index test and the reference standard are about the way in which diagnostic tools can classify and detect the disease.

Two independent reviewers (DS-V and AM-V) applied QUADAS-2 tool to the studies included in the systematic review, filling out a summary table with the results of the risk assessment. Discrepancies between the application of the QUADAS-2 tool were pooled and resolved through discussion between the two reviewers, and the Kappa-Cohen Index was calculated to examine the inter-operator agreement with SPSS 24 Statistics (IBM) with a significance level of 95% (*p* < 0.05).

## 3. Results

### 3.1. Study selection

**Figure 2:**
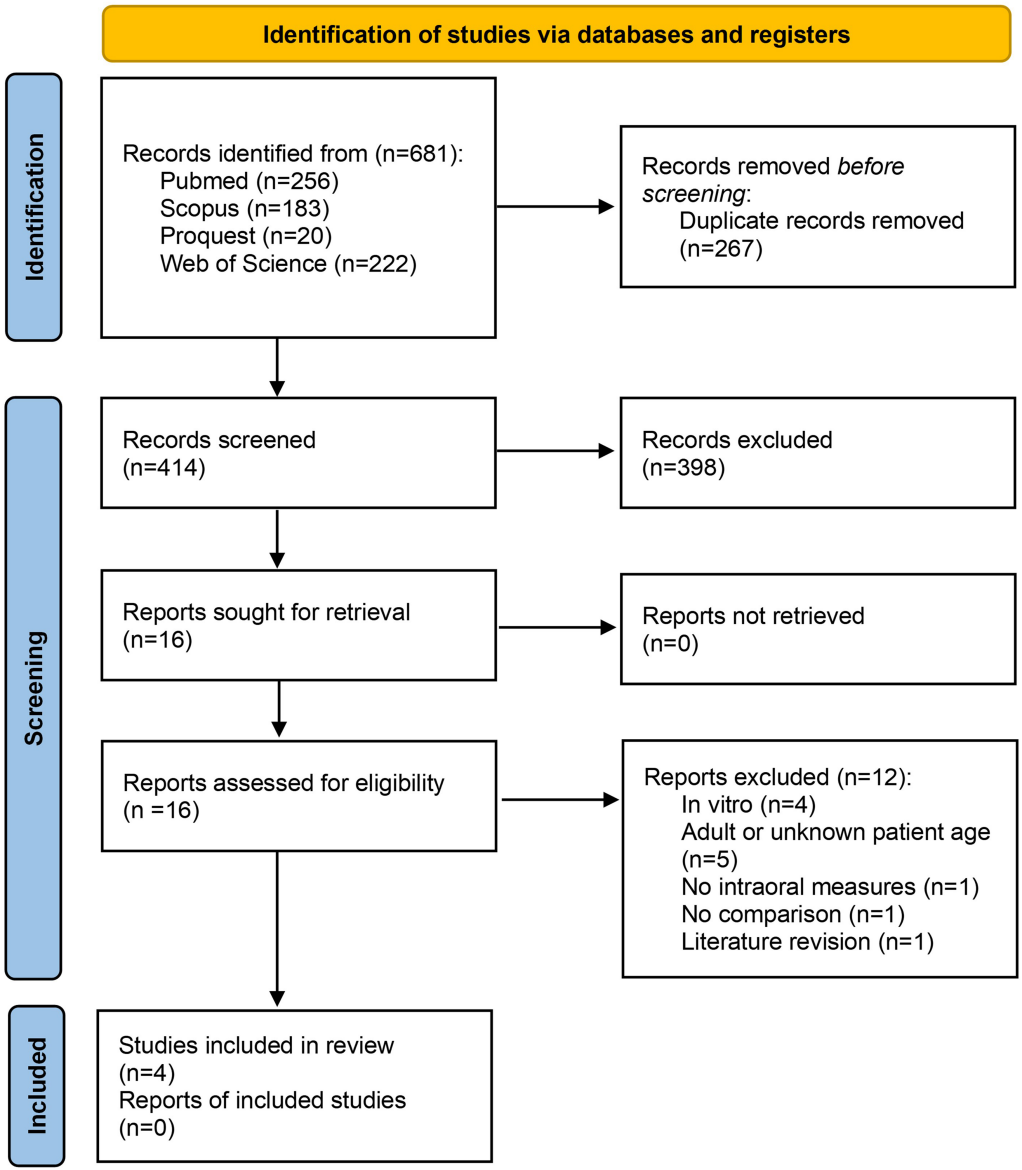
Identification of studies via databases and registers, adapted from PRISMA (15).

Initially, 661 articles were obtained through the search in databases (Table 1). After the article selection process following PRISMA guidelines (Figure 2), a total of four articles meeting the inclusion-exclusion criteria were obtained (Table 2). 12 of the studies were eliminated in the final phase after reading the entire article because they corresponded to *in vitro* studies or did not meet the proposed age range, among others. We searched in the reference lists of the selected full-text studies, but no record was obtained that had not been previously included, so this item was not included in the PRISMA diagram. The results of the Kappa-Cohen index for the analysis of the inter-operator agreement were between substantial and almost perfect for both the Risk of Bias (*κ* value 0.6–1) as for Applicability Concerns (*κ* value 0.692–1).

**Table 1:** Search strategy and results ordered by database.

Database	Search strategy	Results
PubMed	((digital impression) OR (intraoral digital impression) OR (intraoral scanning) OR (intraoral scanner) OR (intraoral digital scanner)) AND ((child*) OR (pediatric)) NOT (adults)	256
Scopus	((TITLE-ABS-KEY (digital AND impression) OR TITLE-ABS-KEY (intraoral AND digital AND impression) OR TITLE-ABS-KEY (intraoral AND scanning) OR TITLE-ABS-KEY (intraoral AND scanner) OR TITLE-ABS-KEY (intraoral AND digital AND scanner)) AND (TITLE-ABS-KEY (child*) OR TITLE-ABS-KEY (pediatric)) AND NOT TITLE-ABS-KEY (adults))	183
ProQuest	((digital impression) OR (intraoral digital impression) OR (intraoral scanning) OR (intraoral scanner) OR (intraoral digital scanner)) AND ((child*) OR (pediatric)) NOT (adults)	20
Web of Science	((digital impression) OR (intraoral digital impression) OR (intraoral scanning) OR (intraoral scanner) OR (intraoral digital scanner)) AND ((child*) OR (pediatric)) NOT (adults)	222
TOTAL		**681**

The bold value is total results of the data search.

**Table 2:** QUADAS-2 judgements for quality assessment of the included studies.

Study	Risk of bias				Applicability concerns		
	Patient selection	Index test	Reference standard	Flow and timing	Patient selection	Index test	Reference standard
Patient perception							
Burdhardt et al. (8)	Low risk	Low risk	Low risk	Low risk	High risk	Low risk	Low risk
Glisic et al. (9)	Unclair	Low risk	Low risk	Low risk	High risk	Low risk	Low risk
Yilmaz and Aydin (10)	Unclair	Low risk	Low risk	Low risk	High risk	Low risk	Low risk
Accuracy							
Glisic et al. (9)	Unclair	Unclear	Low risk	Low risk	High risk	Low risk	Low risk
Liczmanski et al. (12)	Low risk	Unclear	Unclear	Unclair	Low risk	High risk	Unclair
Chairside time							
Burdhardt et al. (8)	Low risk	Unclear	Unclear	Low risk	High risk	Unclear	Unclear
Glisic et al. (9)	Unclair	Low risk	Low risk	Low risk	High risk	Low risk	Low risk
Yilmaz and Aydin (10)	Unclair	Low risk	Low risk	Low risk	Hig risk	Low risk	Low risk

The articles finally selected, and their characteristics can be seen in Table 2. The total number of studies in each category were three for the analysis of the patient's perception and scanning or impression time; and two items to assess the reliability and/or reproducibility of intraoral scans. The quality of the publication was analyzed for each field evaluated.

### 3.2. General characteristics and main demographics of studies included

All included studies have a repeated measures design. From the methodological point of view, it is transversal since although the data collection is carried out on separate dates to avoid bias, time does not act as a variable, temporary changes are not analyzed. Yilmaz and Aydin (10), Liczmanski et al. (12) and Glisic et al. (9) followed the same sequence of printing and scanning, without creating groups or randomizing. Burhardt et al. (8) designed their study with parallel, randomized, crossover groups dividing patients into three groups in which they performed scanning with two intraoral scanners and conventional impression in a different order, to avoid patient preference bias.

Regarding the geographical location of the study, it was different in all cases, being Turkey (10), Germany (12), Netherlands (8) and Denmark (9). However, it is considered that the location of the study is irrelevant for the study since it does not affect the reliability or perception of the patients before a measurement instrument. With respect to the sample size used, Yilmaz and Aydin (10) have a sample size of 28 children, Liczmanski et al. (12) of 26 children (44 dental arches) and Glisic et al. (9) of 59 children. Burdhart et al. (8) included 38 children without sample size calculation. The mean age of the patients ranged from 9.3–12.7 years of age. Regarding the intraoral scanners used, they were Lava C.O.S (3M ESPE, St Paul, Minn) and Cerec Omnicam (Sirona Dental Systems, Bensheim, Germany) (8), TRIOS Classic (Version 1.4.6.0. 3Shape, Copenhagen, Denmark) (9), TRIOS 3-Cart (Color-2017, 3shape, Denmark) (10) and TRIOS Ortho (3Shape, Copenhagen, Denmark) (12). All the intraoral scanners analyzed work with intraoral cameras that obtain the three-dimensional image through a direct video sequence, immediately transforming it into 3D format. The Cerec Omnicam System (Sirona Dental Systems, Bensheim, Germany) uses active triangulation technology with white led light, while TRIOS (3Shape, Denmark) uses confocal laser scanning (unrevealed light source) and Lava C.O.S (3M ESPE, St Paul, Minn) active wavefront sampling with pulsed blue light. Regarding the System, Cerec and Lava C.O.S. they require a layer of powder to perform an adequate intraoral scan, while the rest of the systems belonging to the 3 Shape TRIOS range scan directly.

### 3.3. Quality assessment of selected studies

**Figure 3:**
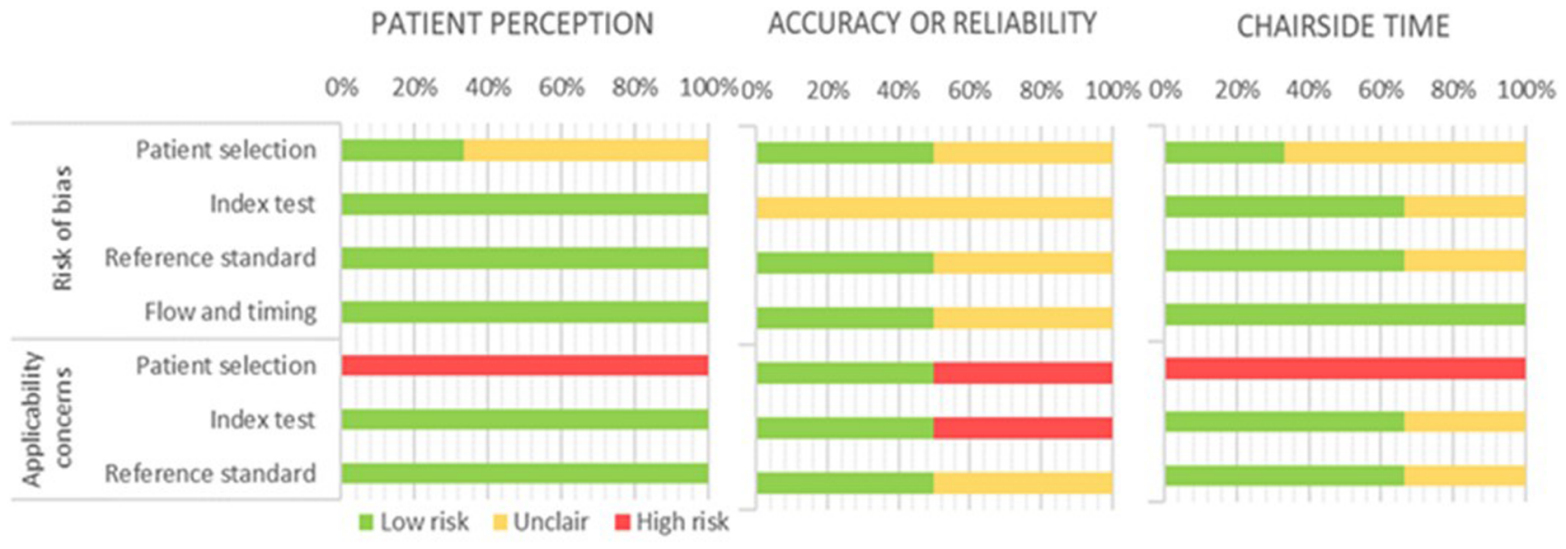
QUADAS-2 judgements for quality assessment of the included studies.

The quality of all selected studies in the review was assessed using the QUADAS-2 tool (Table 2 and Figure 3).

In the risk of bias analysis of patient perception, only one of the three studies had a low risk of bias (8) in terms of patient selection, although all of them had a high risk in terms of study application; the indices used, the standard reference and the flow of the study were considered low risk and adequate applicability. Regarding the risk of bias analysis, the only study that is considered low risk is Burdhart et al. (8), however, its applicability is low since it only includes children 10–17 years old, excluding younger age groups. Both Glisic et al. (9) and Yilmaz et al. (10) present an indeterminate or moderate risk, since they do not establish how they carry out the sampling procedure, both being of low applicability; Glisic et al. (9) select children between the ages of 9–15 years and with severe malocclusion, leaving out a large percentage of younger normoclussive patients, and Yilmaz et al. (10) present children of very similar ages. The indices used to assess the patient's perception (questionnaires), the reference standard used, and the study flow were adequate in all cases. We have considered that if the study is in the pediatric population, children of all age groups should be selected, since taking children of adolescent age cannot subsequently extrapolate the results to the general pediatric population. For this reason, studies that leave out a large part of their target population have been characterized as having low applicability.

All studies assessed had unclear risk of bias and low applicability of results in terms of accuracy or reliability. In the study by Glisic et al. (9) the intraoral measurements made are perfectly specified for both procedures, as well as the measurement instrument, however, the patients included by age would be in mixed dentition, and it is not specified what happens in case that the canines are not present to carry out the measurements, therefore, although the method is well specified, we consider that the risk of bias is uncertain. Liczmanski et al. (12) presented a non-probabilistic sampling of consecutive cases with inclusion criteria consistent with their objectives and therefore adequate. The measurement instrument and the standard reference have been categorized as unclear, since the measurements are made digitally, and it is not clear if they were all carried out by the same operator blinded to the measurements. In addition, as the measurements have not been made directly on the patient or on the plaster casts, the reliability of the scans of the plaster casts is unknown. Due to this, the measurement method and the standard reference have been considered of low and uncertain applicability, respectively.

Regarding the chairside time, the risk of bias and the applicability of the results of the collected studies were analyzed. There was a 66.7% uncertain risk of bias in the articles with respect to the selection of patients, and 33.3% with respect to the index and the standard reference used, while the risk of bias in terms of study flow and time was low in all the selected studies. The study by Burdhart et al. (8) was of moderate risk of bias, since, although the selection of patients and the flow of the study were of low risk of bias, the index and reference tests were of indeterminate risk since they do not specify the methods of evaluating the time of scanning or printing. In addition, adding to the previously mentioned fact of the low application to the selected population, while their objective was to evaluate the perception of time, they evaluate time as a measurement, so they do not correspond and therefore the applicability decreases. The studies by Glisic et al. (9) and Yilmaz and Aydin (10) are considered of uncertain risk but low applicability in terms of patient selection, as already mentioned. The index and reference tests of both authors have a low risk of bias, since both procedures are well described and specified, so their applicability is high.

### 3.4. Main outcomes

The main outcomes of the included studies in this systematic review are summarized in Table 3.

**Table 3:** Main characteristics and outcomes of the studies included in the systematic review.

Study	Population				Material and methods					Main conclusions
	Sample size	Age (years)		Inclusion-exclusion criteria	Intraoral scanner	Compared methods	Assessed parameters	Recorded measurements	Statistical analysis	
	*n*	Range	Mean							
Burdhardt et al. (8)	38	10–17	12	No previous experience with alginate impressions or IOS, good physical and mental health, no craniofacial disorders.	- Lava C.O.S. - Cerec Omnicam	Alginate impression	Patient perception and preference	- Perception questionnaire (Likert scale) - Preference questionnaire	Friedman test, Wilcoxon signed rank test, Spearman correlation test.	Young orthodontic patients preferred the digital impression over alginate, although alginate impressions required the shortest chairside time
							Time	- Total chairside time		
Glisic et al. (9)	59	9–15	12.7	9–15 years-old, no previous orthodontic treatment or experience with alginate impressions or IOS, no craniofacial syndromes or general diseases.	TRIOS Classic	Intraoral measurements and plaster casts from alginate impression	Patient experience questionnaire and accuracy	- Anxiety questionnaire (VAS) - Comfort questionnaire	Student *T*-test, exact test based on binomial distribution, General Linear Model, paired *t*-test.	Patient experience was better during IOS compared with alginate impression, although there were not differences in chairside time. Dental arch distances on digital models and plaster casts were larger compared with the intraoral measurements, but there were no differences between models and plaster casts.
							Accuracy	- Intercanine distance - Intermolar distance - Canine-molar distance		
							Time	- Total chairside time		
Yilmaz and Aydin (10)	28	7.08–12.92	10.16	Patients who needed impression, no history of digital or conventional impression, no TMD or periodontal discomfort, not to be using psychiatric or neuropathic drugs.	TRIOS 3-Cart	Alginate impression	Patient comfort and preference questionnaire	- Comfort index modified for children (VAS) - Preference questionnaire	Student *T*-test, *U* Mann–Whitney test, Pearson's correlation coefficient test.	Both conventional and digital impressions were similar in terms of the time required to take impressions. The digital impression method was more comfortable. Most of the children preferred the digital impression method.
							Time	- Total impression time		
Liczmanski et al. (12)	26	–	9.8–9.3 (girls-boys)	Orthodontic patients in active treatment, patients in mixed dentition phase, need of alginate impression for an orthodontic appliance, no handicapped patients with oral sensomotory discomfort or restricted mouth opening capabilities.	TRIOS Ortho	Digital models from plaster models (alginate impressions)	Accuracy	- Number of polygon points (overlap) - Total absolute deviation from digital models	Paired Student *t*-test, Wilcoxon test, Kruskal–Wallis test or Mann–Whitney test.	Dimensional differences between IOS and conventional alginate impressions in the mixed dentition are clinically tolerable. In all clinical situations of active treatment in the mixed dentition, the IOS is more detailed and less error-prone.
							Scanning time	- Total scanning time		

TMD, temporomandibular disorder; VAS, visual analog scale; IOS, intraoral scanner.

#### 3.4.1. Patient perception

Three studies were selected that evaluated the perception of the patient (8–10). In a study conducted with CEREC and Lava C.O.S. by Burdarht et al. (8) the gag reflex, breathlessness and time perception were found to be similar between digital scanning and conventional impressions, but children were significantly more nauseated (*p* = 0.00) and less comfortable (*p* = 0.02) with alginate impressions than with digital impressions in the maxilla. 51% of children preferred intraoral scanning, followed by 29% preferring conventional impressions and 20% children with no preference. Glisic et al. (9) carried out a study with TRIOS Classic, obtaining a significantly better experience with the intraoral scanner than with the alginate impression in terms of comfort (*p* < 0.05), except for the perception of time (*p* = 0.368) and temperature (*p* = 0.4259), which were similar for both procedures. In the study by Yilmaz and Aydin (10), they found significantly greater discomfort with conventional printing compared to digital with TRIOS 3-Cart, both perceived by the clinician and by the child (*p* < 0.001 for both comparisons).

#### 3.4.2. Accuracy or reliability

Two articles were found that evaluated the reliability of intraoral scanning measures vs. impressions. Glisic et al. (9) state that the intraoral measurements between the upper canines were significantly smaller than in the stone models (mean difference −0.2 mm) or digital models (mean difference −0.2 mm) obtained with TRIOS Classic. The intraoral measurements obtained between the maxillary first permanent molar and canine were also smaller than in the stone models (mean difference −0.28 mm) or the digital models (mean difference −0.36 mm). On the other hand, the differences found between the plaster casts and the digital models were not significant, and therefore similar in all the measurements (upper and lower intercanine distances, upper and lower intermolar distances, and distances between canine and ipsilateral molar). In another study carried out with TRIOS Ortho (12) in which intraoral scanners and images obtained by scanning the stone model were evaluated three-dimensionally, obtaining an absolute mean difference of 0.022 mm (SD = 0.027) between the digital models obtained directly from the patient and by scanning the plaster model, with no significant effect of gender, age, scanned arch, or type of malocclusion (*p* > 0.05).

#### 3.4.3. Chairside time

Three articles that evaluated scanning or impression time were selected (8–10). Significantly longer chair time (*p* < 0.02) is observed with the Lava C.O.S. intraoral scanner. (17.83 min, SD = 3.99) than with CEREC Omnicam or conventional impressions according to Burdhart et al. (8), obtaining similar times between the traditional method (mean = 9.72 min, SD = 1.81) and CEREC Omnicam (mean = 10.74 min, SD =1.81). No significant differences (*p* = 0.916) were found in patient chair time (9) between conventional (mean = 11.92 min, SD = 4.62) and digital printing with TRIOS Classic, (mean = 12.08 min, SD = 6.55) for intraoral scanning. In a study (10) of the time between TRIOS 3-Cart and conventional printing, it was determined that there were no differences in the total time spent in the two procedures (*p* = 0.41). However, the authors found a significantly longer time spent for conventional impression (mean = 167.11 sec, SD = 48.18) than with intraoral scanning (mean = 138.39 sec, SD = 26.08) in the upper arch (*p* = 0.008), but a longer time for bite registration with the intraoral scanner (mean = 106.75 sec, SD = 34.24) than with the alginate impression (mean = 71.29 sec, SD = 17.88) (*p* < 0.001).

## 4. Discussion

Full-arch intraoral recording with intraoral scanners has become popular in recent decades, reporting adequate accuracy (18, 19), being very useful in orthodontic specialty (20). However, most of these studies are conducted in adult patients, while a large percentage of the volume of orthodontic patients are children and/or adolescents. After the results obtained in the search in the databases, it is relevant that many of the studies had to be excluded from the analysis because they were *in vitro* studies (11, 21–24) or with adult subjects or not reported age of the patients (4, 5, 25–27). Some of the studies have resulted in low population extrapolation because they have studied children in the adolescent stage, ignoring younger children who are precisely the ones that present the most behavioral problems during impressions. In the time lapse between the bibliographic search and the analysis of the variables and the bias of the studies, two articles have been published that deal with intraoral scanners in children. Of them, one is an *in vitro* study (28), therefore, it would not have been included according to our inclusion-exclusion criteria. The other article (29) is a clinical trial, which is why it will be discussed in this section.

Regarding the perception of the patient, in studies without age restriction (4, 30) it was determined that there are significant differences between the traditional method (alginate) and intraoral scanners (iTero and TRIOS) in patient comfort, perception of time, sensation of pain or dry mouth. In a recent systematic review, the experience, and preferences of patients of all ages with intraoral scanners were evaluated, finding a better overall experience with the digital method compared to the analogue method, especially in terms of taste, smell, sound, vibration, nausea, and queasiness; however, no differences were found in the level of anxiety of the patients (31). Perception is described as the grasp of reality; the studies analyzed study the perception as comfort of the patients, analyzing variables such as sensation, breathing, taste and smell, thermal sensation, nausea or gag reflex, pain, sound, or nervousness (8–10). The data obtained in our research coincide with previous authors, confirming that in children there is also a feeling of discomfort and more nausea (8–10, 29) in conventional alginate impressions. However, the children did not report significant differences in terms of perception of time (8, 9), temperature (9) or pain (29).

In general, a preference of the subjects to prefer the digital method over the conventional one has been observed, regardless of the age analyzed (4). Most children prefer intraoral scanning (8, 10) being an 89.3% of children who reported feeling stressed with the conventional method, compared to 3.6% with the digital method (10). The differences in preference recently reported by Bosoni (29) are lower (75% and 25% respectively for the digital and conventional method), but in agreement with previous studies. This contrasts with the results of Grünheid et al. (32) since the authors report that the patients (both adults and children) in their study continued to prefer the conventional method to the digital method due to the shorter time required. In our opinion, the age difference of the study sample may be decisive for this difference, since children have smaller oral cavities, more difficult to make impressions and may present more discomfort as a result. In addition, adult patients have been subjected to more impressions throughout their lives, being more accustomed to the procedure, while children are often their first impression.

In relation to the reliability or accuracy, we found two systematic reviews carried out without age restriction. The results of these systematic reviews established the lack of evidence regarding the reliability of both procedures since the study designs and their methodology were of great variability (6). Besides, the authors considered that there is limited evidence about the equivalences obtained in the inter- and intra-arch measurements of digital models obtained from intraoral scanners vs. the stone models or the images generated from conventional prints (33). In addition, there is evidence that while the digital iTero models are highly reliable and reproducible, the printed models of these digital models present dimensional differences (25). The factors that influence the accuracy of intraoral scanners have been studied, but the evidence is poor, since it depends on the scanning system used, the operator's experience and the impression material (34, 35). However, there is evidence that full-arch scans are more susceptible to deviation, with smooth, regular surfaces being the easiest to capture with the intraoral scanner (36). In addition, it has been observed that full-arch intraoral scanning provides less precision than extraoral digitalization of the model obtained, since factors such as the amount of saliva or intraoral space influence (37). In a study conducted in adults (38) with five scanners (CEREC Omnicam, CS600, iTero, TRIOS 3 and i500) it was determined that dimensional reliability depends on the scanner used, with CEREC Omnicam being the one that showed the most dimensional errors. There are only two studies that analyze the reliability and/or accuracy in children. A magnification is found in the digital models scanned with TRIOS Classic compared to the intraoral measurements in the maxillary intercanine distances and between the ipsilateral molar and canine (9). In another study carried out with TRIOS Ortho, a difference of 0.022 mm was found between the digital model and the digitization of the stone model (12), suggesting a minimal variation between both reproductions.

In studies without age restriction, it has been observed that the intraoral scanning time can be significantly higher (4) or lower (30) than conventional alginate impressions, depending on the intraoral scanner used and the training of the operator; however, by adding the working time in the dental chair and the processing time, the total times of both procedures are equalized (32). The results in terms of scanning time vs. impression time in children are not consistent. Only two studies report statistically differences in the comparison between the digital and conventional method, stating that working time is higher with Lava C.O.S. scanner (8) or shorter with TRIOS 3 (29) than with the alginate-traditional method. On the other hand, some studies report that there are no significant differences scanning with CEREC Omnicam (8), TRIOS Classic (9) or TRIOS 3-Cart (10) compared to the traditional method. Observing the examined arches in a different way, the intraoral scanning time is significantly shorter in the upper arch registration than with the conventional method, probably due to the gag reflex and the arches produced by the alginate. Regarding the perception of time by the patients, 64.3% of the children thought that the conventional method lasted longer than the intraoral scan (10), although other authors didn't find differences (8) despite real differences in working time.

Regarding whether the type of dentition or malocclusion affects the intraoral scanning, in an *in vitro* study evaluating digital models from intraoral scanning and digital models from the digitalization of the plaster models with TRIOS Ortho, the authors state the absence of differences between patients in early and late mixed dentition and Angle type I, II or III malocclusion with respect to absolute total deviation or scan time (12).

Although the digital registration method is much more expensive than the traditional one initially, the costs equalize after three years (9) or less since the repetition of impressions is avoided, the cost of the auxiliary staff of the clinic, the space of the laboratory, among others, so the determinant for the authors is the frequency of taking impressions in the dental clinic (39).

The evidence about the limitations of intraoral scanners seems to confirm the higher learning curve that they require compared to conventional impression taking and stone casting. Additionally, liquids such as saliva or crevicular fluid can cause registration errors due to optical refraction (40). Considering the use of intraoral scanners in pediatric patients, the smaller size of their oral cavities is added, in addition to the noises and/or sensations produced by the optical head; although these also complicate conventional impression taking compared to adults.

This study has certain limitations. On the one hand, the patient's perception should be evaluated with parallel groups and cross design to avoid the bias in relation to the order of the procedures. On the other hand, data collection based in direct survey or visual-analogue scales (VAS) in children depends on the level of maturity of each child, and could be not reliable. In addition, the measurements used to evaluate reliability or accuracy are not the same in the different studies, so extrapolation of results must be done cautiously, since as discussed, there are slight differences between them.

Despite these limitations, this systematic review has the strength of being the first conducted in children. As is foreseeable, the pediatric patient presents differences with respect to the adult patients that make us change our way of working with them, in terms of the size of the oral structures, maturity, and behavioral aspects. As seen in this research, conventional alginate impressions are a stressful procedure, especially in pediatric patients, in which the introduction of impression material into the oral cavity for a few minutes can be very difficult. Therefore, due to the absence of clinically relevant differences in terms of the reliability of the reproduction of the dental arches, we must consider the use of intraoral scanners in children as a very favorable option, reducing discomfort for patients and obtaining advantages for the dentist such as the absence of physical storage space, no time to empty the plaster and savings in impression and printing materials despite the high initial costs.

After analyzing the results of our research and the existing literature, we believe in the need to create reliability clinical studies of intraoral scanners with parallel crossed groups that evaluate the perception of children, as well as the accuracy with respect to the patient's intraoral measurements of the digital model and printed models compared to the gold-standard (plaster models). In addition, in most of the studies analyzing intraoral scanners vs. the conventional method, the associated costs, the actual total time required, or the preferences of the patients (and not only their experience) are not considered (33). Alginate impressions and stone models have been considered as the gold standard in reproduction of intraoral structures, but these must be evaluated with respect to the new digital methods in comparison with the direct measurements of the patient, since they also present dimensional deviations.

## 5. Conclusions

The use of intraoral scanners in children is a favorable option, finding a significantly higher patient perception and comfort with intraoral scanners compared to the conventional impression method. The evidence for reliability or reproducibility is not strong to date, however, the differences between the intraoral measurements and the digital models would be clinically acceptable.

## Data Availability

The raw data supporting the conclusions of this article will be made available by the authors, without undue reservation.
